# Differential and Combined Effects of Physical Activity Profiles and Prohealth Behaviors on Diabetes Prevalence among Blacks and Whites in the US Population: A Novel Bayesian Belief Network Machine Learning Analysis

**DOI:** 10.1155/2017/5906034

**Published:** 2017-08-27

**Authors:** Azizi A. Seixas, Dwayne A. Henclewood, Aisha T. Langford, Samy I. McFarlane, Ferdinand Zizi, Girardin Jean-Louis

**Affiliations:** ^1^Department of Population Health, Center for Healthful Behavior Change, NYU School of Medicine, 227 East 30th Street, New York, NY 10016, USA; ^2^Booz Allen Hamilton Inc., Boston, MA, USA; ^3^Division of Endocrinology, Department of Medicine, State University of New York (SUNY) Downstate Medical Center, Brooklyn, NY 11203, USA

## Abstract

The current study assessed the prevalence of diabetes across four different physical activity lifestyles and infer through machine learning which combinations of physical activity, sleep, stress, and body mass index yield the lowest prevalence of diabetes in Blacks and Whites. Data were extracted from the National Health Interview Survey (NHIS) dataset from 2004–2013 containing demographics, chronic diseases, and sleep duration (*N* = 288,888). Of the total sample, 9.34% reported diabetes (where the prevalence of diabetes was 12.92% in Blacks/African Americans and 8.68% in Whites). Over half of the sample reported sedentary lifestyles (Blacks were more sedentary than Whites), approximately 20% reported moderately active lifestyles (Whites more than Blacks), approximately 15% reported active lifestyles (Whites more than Blacks), and approximately 6% reported very active lifestyles (Whites more than Blacks). Across four different physical activity lifestyles, Blacks consistently had a higher diabetes prevalence compared to their White counterparts. Physical activity combined with healthy sleep, low stress, and average body weight reduced the prevalence of diabetes, especially in Blacks. Our study highlights the need to provide alternative and personalized behavioral/lifestyle recommendations to generic national physical activity recommendations, specifically among Blacks, to reduce diabetes and narrow diabetes disparities between Blacks and Whites.

## 1. Introduction

The prevalence of dysglycemia, prediabetes, and diabetes among blacks is almost double the rate among whites (13.2 versus 7.6% in Blacks and Whites, resp.). Furthermore, Blacks have poorer glycemic control and higher diabetes-related mortality and morbidity such as amputations, renal disease, and diabetes-related blindness [1–7]. Biology, community/neighborhood, poor or limited healthcare, and behavior/lifestyle are all potential contributors to this disparity [8]. Of the possible causes for this disparity, behavioral/lifestyle factors (e.g., diet, exercise/physical activity, inadequate podiatry care, and monitoring blood glucose levels) are considered the most proximal and easily modifiable. Specifically, exercise/physical activity might provide the most effective population health benefits for diabetes [9] as the Center for Disease Control and Prevention (CDC) estimates that 15–30% of individuals with prediabetes will develop T2D within five years if they do not engage in regular moderate-vigorous physical activity and lose weight.

Despite the universal health benefits of exercise/physical activity in diabetes prevention and management, Blacks compared to Whites are less likely to adhere to the United States Department of Health & Human Services (USDHHS) recommendations of at least 150 minutes of moderate-vigorous physical activity per week for optimal health benefits [10], and as a result, they may not receive the same health benefits, therefore further widening diabetes disparities between Blacks and Whites [11, 12]. Poor adherence to crude physical activity recommendations, such as the USDHHS recommendations, may be partly due to the lack of personalized and precise interventions. It is therefore likely that differences in exercise/physical activity contribute to differences in diabetes prevalence between Blacks and Whites. However, little is known about which physical activity lifestyles—combinations of quality and intensity (moderate or vigorous), quantity (duration of physical activity), and frequency—could reduce diabetes disparity between Blacks and Whites. Additionally, little is known about the additive effects other behaviors and lifestyle factors (sleep, stress, and body mass index) may have on diabetes prevalence when combined with physical activity, especially among Blacks and Whites.

To address these central questions, we developed a machine learning model, specifically a Bayesian Belief Network machine learning model that utilizes advanced mathematical algorithms that iteratively learn complex relationships and deep insights from big data, using a publicly available national dataset (National Health Interview Survey) to (1) estimate the prevalence of sedentary, moderately active, active, and very active lifestyles; (2) determine the prevalence of diabetes across the different activity lifestyles; (3) determine the prevalence of diabetes across different activity lifestyles between Blacks and Whites; and (4) simulate which physical activity profiles, as well as combination of behaviors and lifestyle factors (sleep, stress, and body mass index), among Blacks would yield a similar diabetes prevalence as Whites. Altogether, we anticipate that our findings will provide more nuanced psychical activity profiles outside of the WHO's guidelines that are associated with low and high diabetes prevalence in Blacks and Whites.

## 2. Methods

### 2.1. Sample

Analysis was based on the data from the 2004–2013 National Health Interview Survey (NHIS) dataset. The NHIS dataset is a nationally representative population-based study of noninstitutionalized adults from 50 US states. Sociodemographic, behavioral, and health conditions and physician-diagnosed chronic disease data were obtained through face-to-face interviews using computer-assisted personal interviewing (CAPI). Interviewers enter participants' responses in CAPI via a laptop computer, which made administration of surveys efficient and improved data quality.

### 2.2. Variables

Variables of interest were derived from a three-step system. First, using PubMed, we reviewed the literature on potential T2D and cardiometabolic risk factors. Second, we compiled a list of T2D and cardiometabolic condition risk factors based on whether data was available for that variable for the years 2004–2013 in the NHIS. Third, the list was winnowed to thirty-four variables, which included (1) target variables—moderate and vigorous physical activity (e.g., leisurely walking/bicycling, slow swimming/dancing, running, lifting weights, and simple gardening activities); (2) sleep duration (short, average, and long); and (3) covariates (sociodemographic, general health, and chronic health conditions, health risk behaviors, and history of physician-diagnosed T2D) (see Table 1).

We acknowledge that our list of T2D risk factors and correlates are not exhaustive, which is partly due to the limited selection of clinical factors and biomarkers in the NHIS. Once the data were retrieved, NHIS sample weights were applied. Additionally, we adjusted the effects of clustering, stratifying, and oversampling for specific population subgroups such as non-Hispanic blacks and individuals who are ±65 years old. After selecting the available variables from the NHIS, the final data set contained 288,888 cases.

#### 2.2.1. History of Diabetes

Individuals were asked whether they ever had a physician who diagnosed them with diabetes.

#### 2.2.2. Moderate Physical Activity

These are activities that “cause only light sweating or a slight-to-moderate increase in breathing or heart rate.” The Field Representative's Manuals for the 1997 NHIS provide examples of light or moderate activities. Examples of moderate physical activity include leisure walking or bicycling, slow swimming or dancing, and light gardening. Participants were also asked about the duration and frequency of their activity.

#### 2.2.3. Vigorous Physical Activity

These are activities that “cause heavy sweating or large increases in breathing or heart rate.” The Field Representative's Manuals for the 1997 NHIS provide examples of vigorous activities. Examples of vigorous physical activity include fast walking, fast bicycling, jogging, strenuous swimming or sports play, vigorous aerobic dance, and strenuous gardening. Participants were also asked about the duration and frequency of their activity.

#### 2.2.4. Sedentary and Active Lifestyles

A sedentary lifestyle was defined as engaging in less than 10 minutes of moderate or vigorous physical activity less than two days per week. While an active lifestyle was defined as engaging in more than 30 minutes of moderate or vigorous physical activity for three or more days per week.

#### 2.2.5. Sleep Duration

Participants were asked “On average, how many hours of sleep do you get in a 24-hour period.” Hours of sleep were entered in whole numbers. Sleep duration reports greater than or equal to thirty minutes (half hour) were rounded up to the next whole number, and those less than or equal to twenty-nine minutes were dropped. For example, total sleep time of 6 hours and 45 minutes was rounded up to a total sleep time of 7 hours, and a total sleep time of 6 hours and 5 minutes was rounded down to a total sleep time of 6 hours.

### 2.3. Machine Learning Analysis

Since traditional regression-based models are sensitive to the effect of confounders, especially in highly complex models, we decided to use BBN machine learning modeling because it is robust to the effects of confounders. BBN machine learning models allow us to investigate highly complex and dimensional networked models and relationships without the interference of confounders and determine hidden insights in big data.

BBN machine learning modeling, unlike traditional regression analysis models, is better at (1) investigating dynamic relationships in big data (data with high levels of volume, velocity, or variety); (2) predictive modeling in determining *Y* in *Y* = *f*(*X*), especially in dynamic modeling and big data; and (3) providing a more accurate explanatory model that captures a model structure that best fits the data, specifically causal inference, as they provide one of the most efficient methods of optimizing data. Its benefits can be revolutionary for the field of medicine as it can yield more accurate and dynamic relationships among multiple factors, a priori and a posteriori [13]. We used the BayesiaLab Version 6.07 statistical software package to construct the current BBN model.

BayesiaLab offers a suite of advanced mathematical and analytical applications predicated on sophisticated algorithms and artificial intelligence that generate structural directed acyclic graphs (DAGs). BayesiaLab can utilize simulation modeling or machine learning modeling of data based on unsupervised and supervised machine learning algorithms that may be employed to derive the conditional probabilities of highly dimensional relationships. The resultant of applying the most appropriate learning algorithm is a directed acyclic graph (DAG), with nodes representing variables and arcs indicative of conditional relationships between nodes. In the resulting DAG, not all nodes may be a part of the learned network. The selected algorithm, the data at hand, and the calculated correlation among the variables will determine whether or not a given node is included in the network. For the current model, we utilized the Tree Augmented Naïve Bayes model algorithm because of its ability to accurately and precisely quantify joint probability distributions and omnidirectional relationships among diabetes correlates and diabetes.

#### 2.3.1. Probability Graphical Models (PGMs) and Conditional Probability Joint Distribution

The BBN machine learning model is a probabilistic graphical model that uses graphical representations to capture the rich independencies and joint distribution of a highly dimensional and complex networked model in space. The independencies and relationships among variables are conditional, and making observational inferences is based on applying conditional evidence to a given node/variable which propagates information throughout the model and updates information on nodes that are conditionally linked—the conditional probability joint distribution.

### 2.4. Preparing the Data

**Figure 1:**
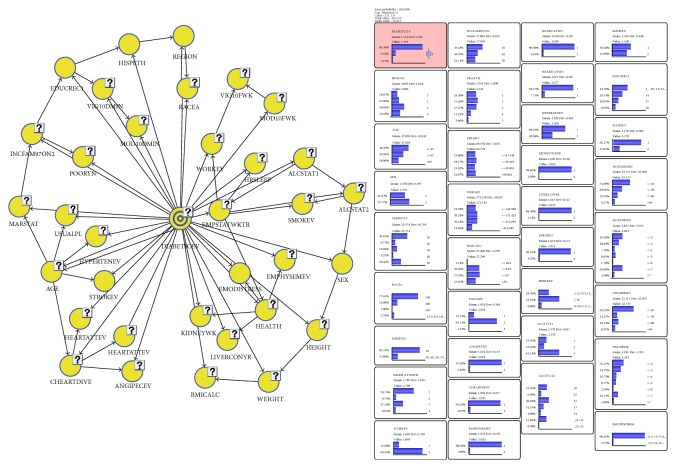
Bayesian Belief Network model of diabetes using the 2004–2013 National Health Interview Survey.

Two of the core phases of data ingestion or preparing data include (1) preprocessing the data to focus on the variables of interest as well as accurately representing valid and missing data entries and (2) binning the data in their most appropriate bins to support the task at hand while remaining in line with the standard practice (see Table 1 for bins and percentage prevalence for each bin). Having the data in the form necessary to support proposed analysis, the data was broken into two, a learning model and test model. The learning model which consisted of 231,111 cases was used as the training data set, while the test model which consisted of 57,777 was used to evaluate how well the BBN model is able to represent data beyond the developed model. After data ingestion, 35 variables/nodes (34 factors and diabetes) were used to build a BBN to investigate the effects of sleep duration and physical activity on diabetes, in the context of demographic, socioeconomic, and general health conditions and health behavior factors (see Figure 1).

### 2.5. Validating the Model and Missing Data

BayesiaLab analytical package was selected for the current study because of its ability to accurately treat missing values through multilevel imputation. Missing values are not uncommon in large population-based data sets like the NHIS. While missing values do represent a lack of information from an incomplete record, retaining incomplete records for subsequent analyses is valuable, and as such, the means of appropriately treating missing values is critical [13].

Once a network has been defined to best represent the data at hand, a series of calibration exercises are then performed to examine the model to ensure that relationships identified by the learning algorithm are valid and intuitive and ensure that the network is not over- or underfitting the data. Once calibrated, the model must then be validated to determine the validity/level of confidence, in order to make inferences from the model [13].

The BBN is an effective modeling tool as it provides a snapshot and high-level overview of relationships underlying the data. Its value lies in its ability to interact with the model and observe changing conditional probabilities, reflecting the impact of the observation of given variable states (bins). Interacting with the model is an effective approach by which to derive observational inference—making inferences based on observation in the collected data. While this level of inference does provide insights into the data and imbedded relationships, such networks lend themselves to further analyses to derive predictive inferences [13].

## 3. Results

### 3.1. Descriptive Statistics

The mean age of the sample was 47.83 ± 18.04 years, and 48.1% were ≤45 years. Of the sample, 77.4% were White; 15.9%, Black/African American; and 6.7%, “other”; 44.3% were male and 55.7% were female; 45.3% reported an annual income less than $35,000 and 24.0% reported an annual family income of $75,000 and higher; 44.76% did not go to college; 83.11% had a usual place where they sought medical care; and more than a third of the sample lived in southern states of the US (36.8%). Regarding health behaviors and conditions, 62.7% were overweight/obese (BMI ≥ 25 kg/m^2^), 19.18% were moderate-to-heavy alcohol drinker, 43.6% smoked more than 100 cigarettes in their lifetime, 2% reported physician-diagnosed chronic kidney disease, and 30.84% reported physician-diagnosed hypertension.

Of the total sample, 51.89% reported less than or equal to 10 minutes of moderate physical activity, 62.42% reported less than or equal to 10 minutes of vigorous physical activity (sedentary lifestyle), 26.81% reported 11–30 minutes of moderate physical activity, 14.27% reported 11–30 minutes of vigorous physical activity (moderately active lifestyle), 15.03% reported 31–60 minutes of moderate physical activity, 16.34% reported 31–60 minutes of vigorous physical activity (active lifestyle), 6.27% reported more than 60 minutes of moderate physical activity, and 6.97% reported more than 60 minutes of vigorous physical activity (very active lifestyle).

Regarding differences between Blacks and Whites, the greatest concentration of Blacks and Whites was in the southern region of the US, which was the only region where Blacks outnumbered Whites. Overall, Blacks (32.54%) had a higher prevalence of hypertension compared to Whites (30.52%). Although the overall diabetes prevalence was 9.34%, Blacks (12.92%) had a higher prevalence than Whites (8.68%) (see Table 1).

## 4. Primary Findings

### 4.1. Sedentary Lifestyle and Diabetes

#### 4.1.1. Sedentary Lifestyle Comparison (Moderate and Vigorous PA)

We investigated the prevalence of diabetes among sedentary Blacks and Whites and found that the prevalence of diabetes among Whites who reported ≤10 mins of vigorous physical activity ≤2 times per week was 11.58%, compared to that among their Black counterparts who had a prevalence of 16.96%.

We observed a similar pattern regarding sedentary individuals who reported moderate physical activity. Among Whites who reported ≤10 mins of moderate physical activity ≤2 times per week, the prevalence of diabetes was 10.10%, while for their Black counterparts, it was 14.90% (see Table 2).

#### 4.1.2. Observational Inference Comparing Sedentary Whites and Blacks

To determine whether increasing the frequency of physical activity per week would significantly lower the prevalence of diabetes among Blacks relative to the average White sedentary individual, we conducted a series of conditional observational inferences where we tested the prevalence of diabetes if an individual reported either 0, 1, 2, 3, 4, 5, 6, or 7 days of moderate or vigorous physical activity. We found that in order for the prevalence of diabetes among sedentary Blacks to be comparable to that among their White counterparts (11.58%), they need to engage in vigorous physical activity 6 days per week (12.32%). For sedentary Blacks who reported some moderate physical activity, in order for them to have a similar diabetes prevalence as Whites (10.10%), they have to exercise 4 days per week (13.51%). Regardless of moderate or vigorous physical activity, the prevalence of diabetes among sedentary Whites is lower than that among their Black counterparts and even among Blacks who exercise more frequently (3, 4, 5, 6, or 7 days per week).

### 4.2. Moderately Active Lifestyle and Diabetes

For moderately active individuals (10–30 minutes of physical activity 4 times/week) who reported some moderate physical activity, the overall prevalence of diabetes was 6.52%, 9.12% for moderately active Blacks and 6.05% for moderately active Whites. For moderately active individuals who reported some vigorous physical activity, the overall prevalence of diabetes was 4.19%, 5.94% for Blacks and 3.88% for Whites. Similar to the results on sedentary individuals, increasing the frequency of physical activity (how many days the individual engaged in moderate physical activity) among Blacks did not lower the prevalence of diabetes to the level of an average moderately active White individual.

### 4.3. Active Lifestyle and Diabetes

The overall prevalence of diabetes among individuals with an active lifestyle (31–60 mins of moderate or vigorous physical activity) was 5.87% (for those who reported moderate physical activity) and 3.11% (for those who reported vigorous physical activity). Active Whites who reported vigorous physical activity 6 days per week had the lowest diabetes prevalence of 2.87%, and for active Blacks, it was 4.42%. Regarding moderate physical activity, active Whites (those who reported 31–60 mins of moderate physical activity 4 days per week) had the lowest diabetes prevalence of 5.44%, while for their Black counterparts, the prevalence was 8.25%. We also found that active Blacks who engaged in greater frequencies of physical activity did not have similar diabetes prevalence as active Whites.

### 4.4. Very Active Lifestyle and Diabetes

For very active individuals who reported moderate physical activity (>60 minutes of physical activity 4 times/week), the overall prevalence of diabetes was 5.99%, 8.41% for very active Blacks and 5.56% for very active Whites. For very active individuals who reported vigorous physical activity, the overall prevalence of diabetes was 3.15%, 4.48% for Blacks and 2.91% for Whites. Similar to previous physically active lifestyles, increasing the frequency of physical activity (how many days the individual reported moderate physical activity) among Blacks did not lower the prevalence of diabetes to the level of the average moderately active White individual.

### 4.5. Physical Activity Profiles Where Blacks and Whites Have Comparable Diabetes Prevalence

Since within-class analyses (only comparing Blacks and Whites with the same physical activity lifestyle profiles) demonstrated that Blacks and Whites had different diabetes prevalence, we tested scenarios in which Whites and Blacks would have similar diabetes prevalence. To do so, we tested across the four different activity lifestyle profiles. We found that in order for Blacks to have a comparable diabetes prevalence to sedentary Whites (<10 minutes of moderate physical activity for less than two times per week with a diabetes prevalence of 8.34%), they had to report 31–60 minutes of moderate physical activity 4 times per week (8.25%). Additionally, we found that in order for Blacks to have a lower diabetes prevalence as moderately active Whites (11–30 mins of moderate physical activity, 5.36%), they had to report vigorous physical activity 4–6 times per week for 31–60 minutes (four times = 4.90%, five times = 4.85%, and six times = 4.42%).

### 4.6. Secondary Findings

Since physical activity alone (regardless of intensity, frequency, and duration) cannot reduce differences in diabetes between Blacks and Whites as evidenced above, we tested which combination of physical activity lifestyles (sedentary, moderately active, active, and very active) and prohealth behavioral/lifestyle factors (body mass index, sleep duration, and stress) reduced diabetes prevalence among Blacks and differences between Blacks and Whites. We found that across all physical activity lifestyle profiles, having normal body mass index (18.5–24.9 kg/m^2^) lowered the prevalence of diabetes the most, followed by sleeping 7-8 hours, then reducing stress levels, and then increasing the frequency of physical activity (see Table 2).

## 5. Discussion

The current study provides new insights into the effects different physical activity lifestyle profiles (sedentary, moderately active, active, and very active) and prohealth behaviors among Blacks and Whites have on diabetes in the United States. First, to our knowledge, our paper is one of the first to pluralize physical activity recommendations—vis a vis the four different physical activity lifestyles—and describe the prevalence of diabetes across these lifestyles among Blacks and Whites. Such an approach decentralizes universal one-size-fits-all health behavior and lifestyle recommendations and offers alternative recommendations for individuals to follow and successfully adhere to. Second, our study attempts to undercut intractable diabetes disparities between Blacks and Whites by offering personalized physical activity profiles that would equally benefit Blacks and Whites, where the two groups would have similarly low diabetes prevalence. Through our findings, it was evident that Whites compared to Blacks had greater diabetes health benefits across the different physical activity lifestyle profiles—sedentary, moderately active, active, and very active—which we discuss below. Third, our study indicates that physical activity alone cannot reduce diabetes disparities between Blacks and Whites based on observational inferences, an inferential approach in Bayesian Belief Network machine learning modeling. We learned that for Blacks, other behavioral strategies, such as healthy sleep, low stress, and normal body mass index (average weight 18.4 to <25 kg/m^2^), need to be supplemented with physical activity to achieve the most health benefits and may provide insights and solutions as to how to reduce diabetes differences between Blacks and Whites. Additionally, we learned which physical activity profiles peered with prohealth behaviors provide the most health benefits in reducing diabetes prevalence among Blacks and Whites. Fourth, our study utilizes novel machine learning analytical models (Bayesian Belief Network modeling) and large population-based data to make observational inferences which can be used to develop personalized and precise behavioral profiles and behavioral recommendations and counselling strategies that might lower diabetes prevalence and reduce diabetes disparities.

Overall, the prevalence of diabetes in our entire sample was 9.34%. Our findings indicate a racial/ethnic difference in diabetes prevalence in the United States, 8.68% for Whites and 12.92% for Blacks. The purpose of the current study was not to find causes of this racial/ethnic difference but rather to explore behavioral solutions (chiefly physical activity) that may lead to lower diabetes prevalence in Blacks and Whites, as well as solutions to reduce diabetes disparity between Blacks and Whites. In general, sedentary lifestyles had no health benefits. Being moderately active, active, and very active had health benefits for Whites regardless of the intensity of activity (moderate or vigorous). While for moderately active Blacks, only those who engaged in vigorous physical activity had significant reductions in diabetes prevalence. However, active and very active Blacks had significant reductions in diabetes prevalence compared to the national levels. Those who engaged in more vigorous physical activity had significant reductions in diabetes compared to those who engaged in moderate physical activity. Ultimately, our findings indicate that there are several physical activity lifestyles associated with diabetes prevalence below the national average, not just the prescribed 150-minutes-per-week recommendation of moderate physical activity.

Our attempt to pluralize and assess alternative physical activity lifestyle recommendations emanated from the growing need to solve the high level of physical activity nonadherence in the United States (52.5% of US individuals do not adhere to Health People 2010 or United States Department of Health & Human Services recommendations) [14]. Although previous studies suggest that behavioral, clinical- and health-related, cognitive and psychological, demographic, environmental, and social factors might contribute to poor adherence to physical activity recommendations, to our knowledge, there has been little attention given to how lifestyle factors compromise an individual's ability to engage in recommended physical activity. Of the little done in this area, there is promising data indicating that racial/ethnic minority women tend to engage in less physical activity due to lifestyle factors such as family needs and child care [15]. Minimizing lifestyle factors that impede adherence to physical activity recommendations might only partially solve the issue and is limited in scope because there are certain lifestyle factors that are not easily modifiable, such as family and work responsibilities. Therefore, providing physical activity recommendations that are accommodating to the heterogeneity of individuals' lifestyles will likely increase overall activity which will decrease diabetes risk and better regulate and manage clinical indicators, such as glucose levels.

Our findings also highlight a possibly inherent racial/ethnic bias in the health benefits of physical activity on diabetes. Some studies have attributed biological, environmental, and lifestyle explanations to the differential benefits of physical activity between Blacks and Whites. Previous studies indicate that Blacks tend to have lower metabolic rates and fat oxidation rates compared to Whites. However, evidence indicating that lifestyle factors such as higher caloric intake and more sedentary lifestyle which are greater in Blacks compared to Whites renders the biology argument inconclusive and highlights the importance of lifestyle factors. Environment also plays an integral role in the relationship between physical activity and diabetes among Blacks and Whites. Rural versus urban settings may affect the impact physical activity has on the prevalence of diabetes [16]. Goedecke and Ojuka argue that the growing urbanization of populations forces individuals to adapt lifestyle behaviors that increase risk of diabetes (such as poor eating habits and physical inactivity) [17]. Additionally, individuals from rural settings are more likely to be physically inactive [18, 19]. Since the majority of Blacks in the NHIS dataset are from rural and urban settings in the United States, it is likely that environment might partially explain why Blacks appear to reap the same benefits as Whites. Therefore, personalized- and precision-based behavioral health approaches—those that deliver targeted and individualized health solutions based on an individual's biology, environment, and behavior/lifestyle—are needed to address racial/ethnic diabetes disparities.

We found that combining other healthful behavioral and lifestyle factors with physical activity may provide additional health benefits for Blacks. Across all physical activity lifestyle profiles, we found that increasing frequency to 4 times per week for moderate physical activity and 6 times per week for vigorous physical activity as well as having low stress was linked to lower diabetes prevalence among Blacks, although not to the prevalence of Whites. Additionally, sleeping 7-8 hours per day on average greatly reduced diabetes prevalence among Blacks than low stress and increasing frequency of physical activity. Overall, having an average body mass index combined with physical activity provided the most health benefits for diabetes, where the prevalence of diabetes was reduced by more than 50% among Blacks across all physical activity lifestyle profiles. Additionally, Blacks who had an average BMI regardless of physical activity profile had a lower diabetes prevalence compared to Whites who had similar physical activity profiles.

The fourth contribution our paper makes is the use of probability-based machine learning analytical and modeling tools (Bayesian Belief Network modeling) to make observational inferences about population health which could be further used to make inferences about individuals and types of individuals. This was evident in our current paper where we were able to determine prevalence of diabetes among different physical activity lifestyles, as well as to test the impact several combinations of behaviors (duration, frequency, and intensity of physical activity, sleep duration, stress levels, and body mass index) have on diabetes and determine which combinations of behavioral/lifestyle health factors lowered the prevalence of diabetes among and between Blacks and Whites. These types of multidimensional and multifactorial observational inferences cannot be done in traditional regression-based models, but through BBN, we are able to circumvent these challenges that fraught traditional regression models. Ultimately, this method brings us closer to determining “what” combination of behaviors may work for “whom”—physical activity lifestyles.

One major limitation with our findings is the inherent bias in physical activity among racial/ethnic minorities. Previous studies found that self-report physical activity varies by race/ethnicity where Blacks/African Americans compared to Whites consistently report lower prevalence of moderate-vigorous physical activity. It is believed that self-report questions about physical activity are culturally and linguistically biased, where Blacks/African Americans consider work and daily activities forms of exercise, and privilege certain organized physical activities that are less common in racially ethnic groups [20]. To avoid these issues, we argue that future studies should utilize both self-report and objective physical activity instruments to accurately determine quantity and quality of physical activity. Although our study utilized advanced mathematical machine learning modeling to test the omnidirectional effects of variables, we were unable to fully parse the confounding effects medical comorbidities have on physical activity and inactivity. Previous studies indicate a dose-response relationship between chronic medical conditions and physical inactivity, and therefore, it is likely that greater levels of medical comorbidity are linked to higher levels of inactivity which increases risk for other medical conditions. In general, distribution of data may affect results; however, we did apply sample weights which we believe may mitigate inferential issues with restricted sample size. However, we do acknowledge that applying multiple evidence to our model in observational inference might reduce the sample size and limit inference, but our large sample size of 288,888 may protect against this.

## 6. Conclusion

Unlike previous studies that emphasized a one-size-fits-all physical activity standard (150 minutes of moderate physical activity), the current study investigated the prevalence of diabetes across four physical activity profiles/phenotypes (sedentary, moderate, active, and very active) among Blacks and Whites in a large population-based dataset in the United States. We argue that such an approach can potentially lead to targeted approaches to physical activity recommendations and audience segmentation. Our secondary findings are that sleep, stress, and body mass index may provide additional health benefits when paired with physical activity for certain profiles of individuals and that this varies by race/ethnicity. Therefore, we highly recommend that diabetes counselling should take a personalized and “bundled approach” where a variety of prohealth behaviors are recommended but personalized to the individual's profile. Moving forward, ubiquitous sensing wearables may be a solution to more accurately assess multiple behaviors without unduly burdening individuals with self-tracking. Data from these wearables can be potentially integrated into electronic medical health records to improve patient-provider communication and shared decision-making around diabetes care. Future studies should assess the acceptability, accuracy, and adherence to wearables by race/ethnicity, physical activity level and profile, and diabetes severity and chronicity (e.g., are wearables more used by newly diagnosed patients or those who are sicker as a result of their diabetes). Lastly, our use of Bayesian Belief Network modeling is novel, and to our knowledge, we are one of the first to use this highly dimensional and robust artificial intelligence machine learning tool, which allowed us to test our primary and secondary questions under different scenarios while taking into consideration the omnidirectional relationships of target variables and confounders in a joint network.

## Figures and Tables

**Table 1:** Differences in sociodemographic, health behavior, and chronic health conditions among Blacks and Whites (*N* = 288,888).

Variables	Total sample (%)	Blacks (%)	Whites (%)
Gender			
Female	55.77	55.62	55.80
Male	44.23	44.38	44.20
Age			
≤45 yrs	48.10	46.69	48.37
46–65 yrs	32.94	33.47	32.84
>65 yrs	18.96	19.84	18.80
Income			
<$35K	45.18	45.57	45.08
$35K–74,999K	30.70	30.59	30.73
>$75K	24.11	23.84	24.19
Education (college)	44.76	44.93	44.64
Usual medical care	83.11	83.59	83.02
Region			
Northeast	16.67	16.22	16.82
Northcentral/midwest	22.00	17.55	23.70
South	36.84	56.29	34.19
West	24.49	9.98	25.29
Weight			
Overweight	37.03	36.92	37.05
Obese	25.67	26.63	25.49
Alcohol			
Moderate	14.19	13.89	14.25
Heavy	4.99	4.87	5.01
CKD	2.00	2.24	1.96
Hypertension	30.84	32.54	30.52
Sedentary lifestyle			
Moderate PA (≤10 minutes)	51.89	52.30	51.81
Vigorous PA (≤10 minutes)	62.42	63.07	62.28
Moderately active lifestyle			
Moderate PA (11–30 minutes)	26.81	26.62	26.85
Vigorous PA (11–30 minutes)	14.27	14.08	14.31
Active lifestyle			
Moderate PA (31–60 minutes)	15.03	14.88	15.06
Vigorous PA (31–60 minutes)	16.34	16.02	16.41
Very active lifestyle			
Moderate PA (>60 minutes)	6.27	6.21	6.28
Vigorous PA (>60 minutes)	6.97	6.83	7.00
Diabetes	9.34	12.92	8.68

*Note*. Alcohol *=* moderate-to-heavy alcohol drinker; CKD = physician-diagnosed chronic kidney disease; hypertension = physician-diagnosed hypertension; overweight = 25 to 29.9 kg/m^2^; obese = ≥30 kg/m^2^.

**Table 2:** Observational inference of physical activity lifestyles and the combined effects of sleep, stress, and body mass index on diabetes in Blacks and Whites.

Profiles	Diabetes total (%)	Blacks with diabetes (%)	Whites with diabetes (%)
*Sedentary lifestyle*			
Moderate PA (≤10 mins of moderate physical activity ≤2 times per week)	10.84	14.90	10.10
Vigorous PA (≤10 mins of vigorous physical activity ≤2 times per week)	12.41	16.96	11.58
Observational inference of combined effects of sleep, stress, and body mass index			
Moderate PA (≤10 mins of moderate physical activity 2 days per week for Blacks)	10.84	14.90 (PA 2 times/week)	10.10
		13.51 (PA 4 times/week)	
		11.74 (PA 4 times/week & 7-8 hrs of sleep)	
		13.08 (PA 4 times/week & low stress)	
		6.48 (PA 4 times/week & BMI = 18.4 to <25 kg/m^2^)	
Vigorous PA (≤10 mins of vigorous physical activity 2 days per week for Blacks)	12.41	16.96 (PA 2 times/week)	11.58
		12.32 (PA 6 times/week)	
		10.69 (PA 6 times/week & 7-8 hrs of sleep)	
		11.92 (PA 6 times/week & low stress)	
		5.87 (PA 6 times/week & BMI = 18.4 to <25 kg/m^2^)	

*Moderately active lifestyle*			
Moderate PA (11–30 minutes, 4 times/week)	6.52	9.12	6.05
Vigorous PA (11–30 minutes, 6 times/week)	4.19	5.94	3.88
Observational inference of combined effects of sleep, stress, and body mass index			
Moderate PA (11–30 minutes, 4 times/week)	6.52	9.12 (PA 4 times/week)	6.05
		7.87 (PA 4 times/week & 7-8 hrs of sleep)	
		8.82 (PA 4 times/week & low stress)	
		4.26 (PA 4 times/week & BMI = 18.4 to <25 kg/m^2^)	
Vigorous PA (11–30 minutes)	4.19	5.94 (PA 6 times/week)	3.88
		5.10 (PA 6 times/week & 7-8 hrs of sleep)	
		5.73 (PA 6 times/week & low stress)	
		2.72 (PA 6 times/week & BMI = 18.4 to <25 kg/m^2^)	

*Active lifestyle*			
Moderate PA (31–60 min, 4 times/week)	5.87	8.25	5.44
Vigorous PA (31–60 mins, 6 days per week for Blacks)	3.11	4.42	2.87
Observational inference of combined effects of sleep, stress, and body mass index			
Moderate PA	5.87	8.25 (PA 4 times/week)	5.44
		7.10 (PA 4 times/week & 7-8 hrs of sleep)	
		7.97 (PA 4 times/week & low stress)	
		3.82 (PA 4 times/week & BMI = 18.4 to <25 kg/m^2^)	
Vigorous PA	3.11	4.42 (PA 6 times/week)	2.87
		3.79 (PA 6 times/week & 7-8 hrs of sleep)	
		4.27 (PA 6 times/week & low stress)	
		2.00 (PA 6 times/week & BMI = 18.4 to <25 kg/m^2^)	

*Very active lifestyle*			
Moderate PA (>60 minutes, 4 times/week)	5.99	8.41	5.56
Vigorous PA (>60 minutes)	3.15	4.48	2.91
Observational inference of combined effects of sleep, stress, and body mass index			
Moderate PA		8.41 (PA 4 times/week)	
		7.25 (PA 4 times/week & 7-8 hrs of sleep)	
		8.13 (PA 4 times/week & low stress)	
		3.91 (PA 4 times/week & BMI = 18.4 to <25 kg/m^2^)	
Vigorous PA	3.15	4.48 (PA 6 times/week)	2.91
		3.84 (PA 6 times/week & 7-8 hrs of sleep)	
		4.33 (PA 6 times/week & low stress)	
		2.03 (PA 6 times/week & BMI = 18.4 to <25 kg/m^2^)	
*Diabetes total sample*	9.34	12.92	8.68

## References

[1] Centers for Disease Control and Prevention (2014). *National Diabetes Statistics Report: Estimates of Diabetes and Its Burden in the United States*.

[2] Herman W. H., Cohen R. M. (2012). Racial and ethnic differences in the relationship between HbA1c and blood glucose: implications for the diagnosis of diabetes. *The Journal of Clinical Endocrinology & Metabolism*.

[3] Kirk J. K., D'Agostino R. B., Bell R. A. (2006). Disparities in HbA1c levels between African-American and non-Hispanic white adults with diabetes: a meta-analysis. *Diabetes Care*.

[4] Spanakis E. K., Golden S. H. (2013). Race/ethnic difference in diabetes and diabetic complications. *Current Diabetes Reports*.

[5] Lanting L. C., Joung I. M. A., Mackenbach J. P., Lamberts S. W. J., Bootsma A. H. (2005). Ethnic differences in mortality, end-stage complications, and quality of care among diabetic patients: a review. *Diabetes Care*.

[6] Zhang X., Saaddine J. B., Chou C. F. (2010). Prevalence of diabetic retinopathy in the United States, 2005-2008. *Journal of the American Medical Association*.

[7] Young B. A., Maynard C., Reiber G., Boyko E. J. (2003). Effects of ethnicity and nephropathy on lower-extremity amputation risk among diabetic veterans. *Diabetes Care*.

[8] Mayberry L. S., Bergner E. M., Chakkalakal R. J., Elasy T. A., Osborn C. Y. (2016). Self-care disparities among adults with type 2 diabetes in the USA. *Current Diabetes Reports*.

[9] Sigal R. J., Kenny G. P., Wasserman D. H., Castaneda-Sceppa C., White R. D. (2006). Physical activity/exercise and type 2 diabetes: a consensus statement from the American Diabetes Association. *Diabetes Care*.

[10] Physical Activity Guidelines Advisory Committee (2008). *Physical Activity Guidelines Advisory Committee Report*.

[11] Shah B. R., Mamdani M., Jaakkimainen L., Hux J. E. (2004). Risk modification for diabetic patients are other risk factors treated as diligently as glycemia?. *Canadian Journal of Clinical Pharmacology*.

[12] Crespo C. J., Smit E., Andersen R. E., Carter-Pokras O., Ainsworth B. E. (2000). Race/ethnicity, social class and their relation to physical activity during leisure time: results from the Third National Health and Nutrition Examination Survey, 1988–1994. *American Journal of Preventive Medicine*.

[13] Conrady S., Jouffe L. (2015). *Bayesian Networks and Bayesia Lab: A Practical Introduction for Researchers*.

[14] Matthews C. E., George S. M., Moore S. C. (2012). Amount of time spent in sedentary behaviors and cause-specific mortality in US adults. *The American Journal of Clinical Nutrition*.

[15] Schoenborn C. A., Stommel M. (2011). Adherence to the 2008 adult physical activity guidelines and mortality risk. *American Journal of Preventive Medicine*.

[16] Richter D. L., Wilcox S., Greaney M. L., Henderson K. A., Ainsworth B. E. (2002). Environmental, policy, and cultural factors related to physical activity in African American women. *Women & Health*.

[17] Goedecke J. H., Ojuka E. O. (2014). *Diabetes and Physical Activity*.

[18] Liu J. H., Bennett K. J., Harun N. (2007). Overweight and physical inactivity among rural children aged 10-17: a national and state portrait. http://rhr.sph.sc.edu/report/SCRHRC_ObesityChartbook_Exec_Sum_10.15.07.pdf.

[19] Patterson P. D., Moore C. G., Probst J. C., Shinogle J. A. (2004). Obesity and physical activity in rural America. *Journal of Rural Health (Spring)*.

[20] Wolin K. Y., Fagin C., Ufere N., Tuchman H., Bennett G. G. (2010). Physical activity in US Blacks: a systematic review and critical examination of self-report instruments. *The International Journal of Behavioral Nutrition and Physical Activity*.

